# Essential Oil of Japanese Cedar (*Cryptomeria japonica*) Wood Increases Salivary Dehydroepiandrosterone Sulfate Levels after Monotonous Work

**DOI:** 10.3390/ijerph14010097

**Published:** 2017-01-21

**Authors:** Eri Matsubara, Yuko Tsunetsugu, Tatsuro Ohira, Masaki Sugiyama

**Affiliations:** Forestry and Forest Products Research Institute, 1 Matsunosato, Tsukuba 305-8687, Japan; yukot@ffpri.affrc.go.jp (Y.T.); otatsu@ffpri.affrc.go.jp (T.O.); sugicchi@ffpri.affrc.go.jp (M.S.)

**Keywords:** *Cryptomeria japonica*, wood, essential oil, salivary biomarkers, DHEA-s

## Abstract

Employee problems arising from mental illnesses have steadily increased and become a serious social problem in recent years. Wood is a widely available plant material, and knowledge of the psychophysiological effects of inhalation of woody volatile compounds has grown considerably. In this study, we established an experimental method to evaluate the effects of Japanese cedar wood essential oil on subjects performing monotonous work. Two experiment conditions, one with and another without diffusion of the essential oil were prepared. Salivary stress markers were determined during and after a calculation task followed by distribution of questionnaires to achieve subjective odor assessment. We found that inhalation of air containing the volatile compounds of Japanese cedar wood essential oil increased the secretion of dehydroepiandrosterone sulfate (DHEA-s). Slight differences in the subjective assessment of the odor of the experiment rooms were observed. The results of the present study indicate that the volatile compounds of Japanese cedar wood essential oil affect the endocrine regulatory mechanism to facilitate stress responses. Thus, we suggest that this essential oil can improve employees’ mental health.

## 1. Introduction

Complex problems such as heavy workloads, poor work conditions, and stressful human relationships in the workplace could promote mental health disturbances. Employee problems related to mental illnesses, including depression, have steadily increased and become a significant social problem in recent years. Work stresses have also been suggested to exert various adverse effects such as obesity, hypertension, and diabetes in workers [1,2,3,4,5]. Medication for several mental illnesses presents a number of unpleasant side effects, including dizziness, nausea, and sleep disturbances. In addition, psychoactive drugs or symptoms of mental illness impair work performance [6,7,8]. Hence, a comfortable workplace and space for relaxation have been suggested to improve or promote mental health.

The effectiveness of several essential oils has been investigated as a strategy to improve the work environment. Essential oil is the aromatic portion of plant constituents; besides imparting a specific flavor and odor most closely associated with the plant itself, essential oils are partly responsible for the pharmaceutical properties of aromatic herbs. Recent studies have discussed the pharmaceutical and therapeutic potential of aromatic herbs, especially in treating symptoms related to mental health, such as anxiety, stress, and depression [9,10]. Research has reported marked reductions in the number of stress symptoms, blood pressure, and heart rate via inhalation of essential oils [11,12]. In addition, several studies have suggested that olfactory stimulation could be useful to ameliorate the fatigue caused by continuous work and improve work performance [13,14,15,16].

Wood is a plant material that has been used since ancient times as a building material. Knowledge of the psychophysiological effects of inhalation of the volatile compounds of wood has grown considerably given new research developments. Several previous reports have indicated that the essential oils of wood or wood chips and their individual volatile compounds could affect the autonomic nervous system when applied to an odorant delivery system controlled by a constant-flow olfactometer [17,18,19,20,21]. However, few studies have investigated the effects of woody odors on psychophysiological responses by focusing on actual spaces for work or relaxation. For instance, Matsubara and Kawai [22] found that volatile compounds emitted in an experiment room constructed of Japanese cedar (*Cryptomeria japonica*) could suppress the activity of the sympathetic nervous system. 

In the present study, we established an experimental method to evaluate the effects of Japanese cedar wood essential oil during and after monotonous work. In Japan, Japanese cedar is the most commonly planted tree in forests, and its timber is often used as an interior decorating material. A calculation task was administered to participants as monotonous work, and salivary stress markers were determined to describe physiological responses before, during, and after the task. We also used questionnaires to achieve subjective assessment of the odor of cedar wood essential oil diffused in the experiment room.

## 2. Methods

### 2.1. Participants and Experimental Design

The experimental design of this study was approved by the Forestry and Forest Products Research Institute and in accordance with the Declaration of Helsinki (2015-FFPRI-876). Nine male volunteers (age: 30.1 ± 6.5 years (mean ± SD); range: 20–39 years), all of whom were clerical officers with daily labor burdens that did not change significantly, were recruited for this research. To avoid the influences of psychological and physiological differences between genders, only males were included in the sample population. None of the participants presented with any abnormality in terms of their physical, mental, or olfactory health or were currently using prescription drugs or were current smokers. While the purpose and schedule of the experiments were explained to the participants, the effects of the wood used in our experiments were not discussed to avoid the influence of individual expectations on the results [23,24]. Written informed consent was obtained from all participants prior to study initiation. 

**Figure 1:**
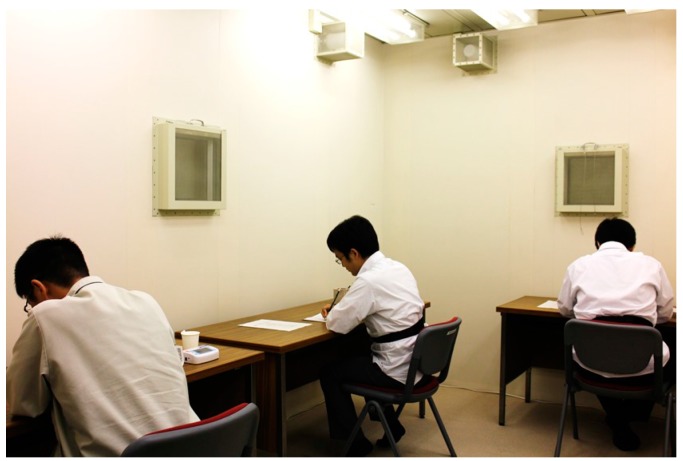
The experiment room in which Japanese cedar wood essential oil was diffused. Three participants entered the experiment room at the same time, sat facing the wall, performed a monotonous task during the work period, and then remained in their seats during the rest period.

**Figure 2:**
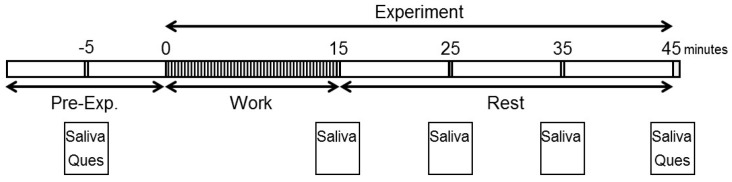
Experimental design. Saliva: saliva sampling, Ques: questionnaire.

Consumption of caffeine was prohibited on the day of the experiment. Wearing strong fragrances and eating food with a strong smell were also forbidden the day before and on the day of the experiment. The experiment room in our research institute (artificial climate chamber, interior dimensions: width, 3000 mm × depth, 4000 mm × height, 2500 mm) was used. The experiment room is shown in Figure 1, and the experimental design is shown in Figure 2. All experiments were conducted between 9:30 and 11:30 a.m. 

Saliva was collected from all of the participants before and four times after they had performed arithmetic work. All of the participants performed the experiment twice at an interval of one week: once in the absence (control condition) and once in the presence (experimental condition) of Japanese cedar essential oil. The order of experimental conditions was counterbalanced between the participants, and none of the participants knew about the room condition prior to the actual experiment.

### 2.2. Preparation of the Essential Oil and Sample for Use with a Diffuser

We used Japanese cedar from Kitayama (Kyoto, Japan) as the experimental material. The material was dried at 45 °C and processed into vertical-grain timber. The timber was then crushed by a wood chipper and hammer mill to produce wood chips. Essential oil was obtained from these chips by steam distillation. To measure psychophysiological effects, we filled a diffuser (Personal Diffuser Squair, At-aroma Co. Ltd., Tokyo, Japan) with a dilute solution containing Japanese cedar essential oil. Several experimenters decided on the optimum concentration of oil in the solution. Finally, 0.45 mL of the essential oil was mixed with 4.05 mL of a dilute solution according to the diffuser instruction manual for diffusion into the experiment room as the experimental condition. Another 4.50 mL of the dilute solution only was prepared for diffusion as the control condition.

### 2.3. Arithmetic Work as Monotonous Work

We used the U-K test [25], which is a serial addition test that requires takers to perform calculations as quickly and accurately as possible. Each participant was supplied with a pre-printed paper containing 15 lines of random, single-digit, horizontally aligned numbers and then instructed to calculate the numbers of a specific line and move to a new line every 1 min. This test was conducted for 15 min.

### 2.4. Subjective Assessment

We used three questionnaires for subjective evaluation of the odor of the experiment room. Irritation to odor was evaluated on a 6-point scale ranging from “not at all” to “strong”. Hedonic responses were rated on a 9-point Likert scale ranging from “extremely comfortable” to “extremely uncomfortable”. A visual analog scale (VAS) was also used for subjective assessment of the odor of the experiment room. This scale consisted of an eight-item questionnaire designed to differentiate subjective responses to the experiment room, including “feel coziness-not feel coziness”, “harmonious-inharmonious”, “friendly-unfriendly”, “can’t concentrate-can concentrate”, “dislike-like”, “artificial-natural”, “uncomfortable-comfortable” and “feel restless-feel calm”. Participants were asked to mark their assessment along a continuous line between each of the two end-points.

### 2.5. Salivary Stress Marker Assay

Salivary α-amylase was measured using a salivary amylase monitor (Nipro Co., Osaka, Japan). The meter tip was immersed in saliva under the tongue of the participant for 30 s and then read within 2 min after collection. We used a cotton swab (Salivette, Sarsteds AG & Co., Nümbrecht, Germany) to measure other parameters. Each participant’s mouth was swabbed for 1 min, and swabs were kept on ice until further processing. Saliva was centrifuged at 1500× *g* for 15 min and then kept at −20 °C until use. Salivary cortisol, interleukin-1β (IL-1β), and dehydroepiandrosterone sulfate (DHEA-s) were measured using enzyme immunoassay (EIA) kits (Salimetrics, State College, PA, USA). Chromogranin A (CgA) was also measured using an EIA kit (Phoenix Pharmaceuticals Inc., Burlingame, CA, USA).

### 2.6. Gas Chromatography-Mass Spectrometry (GC-MS) Analysis

The essential oil was analyzed by a GC-MS system (GCMS-QP2010; Shimadzu Co. Ltd., Kyoto, Japan) equipped with a DB-5 MS capillary column (30 m × 0.25 mm i.d., 0.25 µm film thickness; Agilent Technologies Ltd., Santa Clara, CA, USA). The temperature program was set as follows: 40 °C for 1 min, increased to 180 °C at a rate of 3 °C/min for 2 min, increased to 320 °C at a rate of 4 °C/min, and then held at this temperature for 5 min. We compared the GC-MS data with a mass spectral database library (NIST14) and commercially available reagents for substance estimation.

Volatile organic compounds (VOCs) in the experimental condition of Japanese cedar essential oil were collected using PEJ-02 tubes (Sigma-Aldrich, St. Louis, MO, USA) maintained at 22.5 ± 0.2 °C by applying a flow rate of 0.1 L/min. Collected volatiles were removed from the tubes by heating the trap using an automatic thermal desorption system (TurboMatrix 650, Perkin Elmer Inc., Waltham, MA, USA) at 260 °C for 10 min, cryofocused on a cold trap, and then transferred to a DB-5 MS capillary column (30 m × 0.25 mm i.d., 0.25 µm film thickness; Agilent Technologies Ltd., Santa Clara, CA, USA) for GC-MS analysis (GC 6890/MSD 5973, Agilent Technologies Ltd., Santa Clara, CA, USA). The temperature program was set as follows: 40 °C for 5 min, increased to 180 °C at a rate of 3 °C/min for 10 min, increased to 280 °C at a rate of 4 °C/min, and then held at this temperature for 15 min. We compared the GC-MS data with a mass spectral database library (NIST14) and calculated the concentrations of the target compounds in the sample using β-caryophyllene (Sigma-Aldrich) as the calibration standard.

### 2.7. Statistical Analysis

All of the results are expressed as mean ± SEM. To compare differences among salivary parameters, one-way ANOVA with Bonferroni post hoc test and Student’s *t*-test were performed. For subjective assessment and comparison of work performance between conditions, Student’s *t*-test was used. Statistical significance was recognized at *p*-values of <0.01 or <0.05. All statistical analyses were performed using SPSS 17.0J for Windows (SPSS Japan, Tokyo, Japan).

## 3. Results

To investigate the effects of Japanese cedar wood essential oil after monotonous work, saliva collection, and subjective assessments were performed in this study (Figure 2). VOCs in Japanese cedar wood essential oil diffused into the experiment room were analyzed. During the work period, participants performed arithmetic work and then rested while remaining seated and quiet.

### 3.1. Constituent Analysis of Japanese Cedar Wood Essential Oil in the Experiment Room

We analyzed the VOCs of Japanese cedar wood essential oil by GC-MS and found δ-cadinene, 4-epi-cubebol, cubebol, and several sesquiterpenes as the main components (Table 1). The total volume of volatile compounds of Japanese cedar wood essential oil dissipated in the experiment room was found to be 161.5 µg/m^3^. These compounds were not detected under the control condition.

### 3.2. Analysis of Subjective Assessments

**Figure 3:**
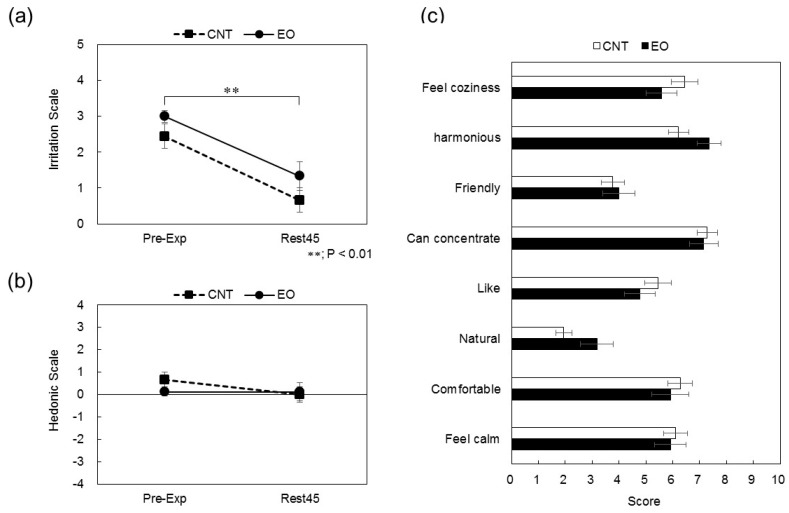
Subjective effects of the odor of the experiment room. (**a**) Irritation scores reported by participants in the control (square) and Japanese cedar wood essential oil (experimental; circle) conditions. CNT indicates the control condition, while EO indicates the experimental condition. Differences in pre-experiment and the end of experiment values were significant in both conditions (*p* < 0.01). Data are shown as mean ± SEM; (**b**) Hedonic scores reported by participants in the control (square) and Japanese cedar wood essential oil (circle) conditions. No significant differences between conditions were observed. Data are shown as mean ± SEM; (**c**) Visual analog scale scores reported by participants in the control (white) and Japanese cedar wood essential oil (black) conditions. Differences between conditions were not significant. Data are shown as mean ± SEM.

Immediately after entering the experiment room, the participants’ responses were rated 3.0 ± 0.2 on the irritation scale and 0.1 ± 0.3 on the hedonic scale for the experimental condition and 2.4 ± 0.3 and 0.7 ± 0.3, respectively, for the control condition. At the end of the experiment, the participants’ responses were rated 1.3 ± 0.4 on the irritation scale and 0.1 ± 0.1 on the hedonic scale for the experimental condition and 0.7 ± 0.3 and 0.0 ± 0.0, respectively, for the control condition (Figure 3a,b). Although time differences were significant (*p* < 0.01) on the irritation scale in both conditions, no significant differences in the hedonic scale were observed between conditions. Levels of the subjective effects of the experiment room were determined by an eight-item questionnaire using a VAS. The VAS was a horizontal line, 100 mm in length, anchored by a word descriptor at each end. The participants marked a point on the line to indicate their subjective response to the experiment room. The VAS score was determined by measuring, in millimeters, from the center of the line to the point marked by the participant. No differences in the VAS scores were observed between the conditions (Figure 3c).

### 3.3. Analysis of Salivary Stress Markers

**Figure 4:**
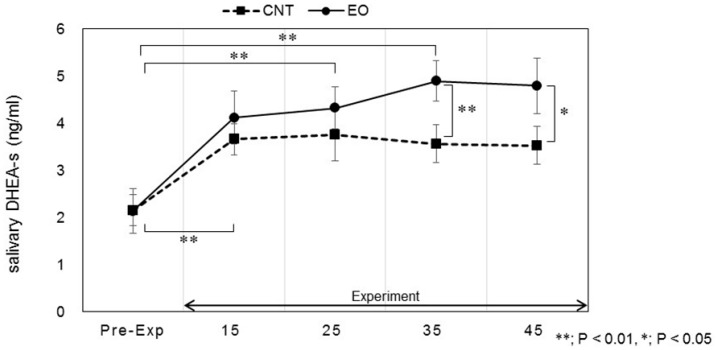
Variation of salivary DHEA-s levels at pre- and during-experiment measurements. The plot in Figure 4 describes changes in the salivary DHEA-s levels of participants in the control (square) and Japanese cedar essential oil (experimental; circle) conditions. CNT indicates the control condition, while EO indicates the experimental condition. Differences observed 35 and 45 min after the calculation task between conditions were significant (*p* < 0.01, *p* < 0.05, respectively). Differences in pre-experiment and during-experiment values between conditions were significant (*p* < 0.01, *p* < 0.05, respectively). ** Statistical significance (*p* < 0.01), * statistical significance (*p* < 0.05). Data are shown as mean ± SEM.

**Figure 5:**
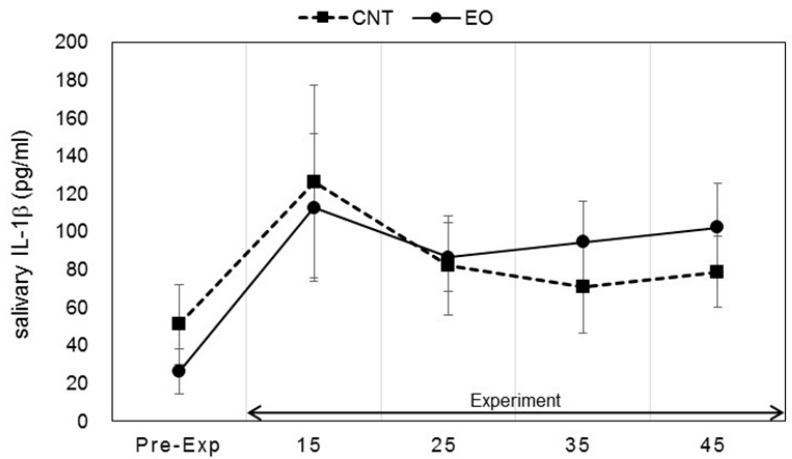
Variation of salivary IL-1β levels at pre- and during-experiment measurements. The plot in Figure 5 describes changes in the salivary IL-1β levels of participants in the control (square) and Japanese cedar wood essential oil (experimental; circle) conditions. CNT indicates the control condition, while EO indicates the experimental condition. Differences between conditions were not significant. Data are shown as mean ± SEM.

**Figure 6:**
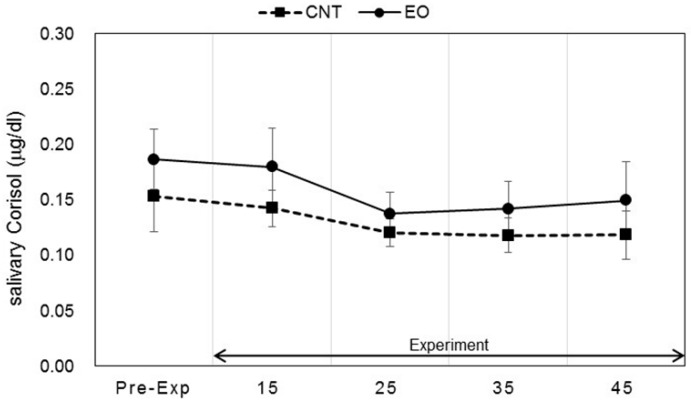
Variation of salivary cortisol levels at pre- and during-experiment measurements. The plot in Figure 6 describes changes in the salivary cortisol levels of participants in the control (square) and Japanese cedar wood essential oil (experimental; circle) conditions. CNT indicates the control condition, while EO indicates the experimental condition. Differences between conditions were not significant. Data are shown as mean ± SEM.

**Figure 7:**
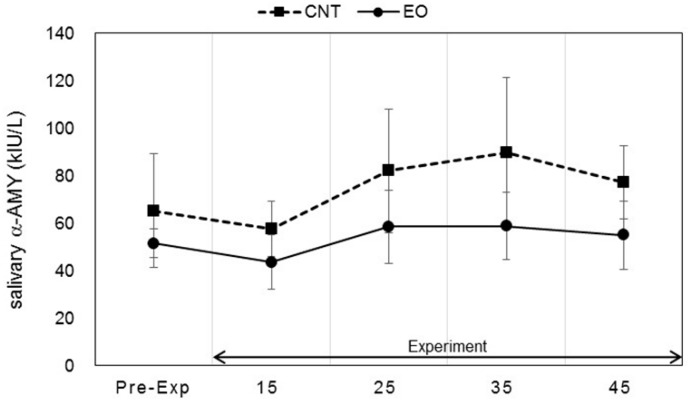
Variation of salivary α-amylase levels at pre- and during-experiment measurements. The plot in Figure 7 describes changes in the salivary α-amylase levels of participants in the control (square) and Japanese cedar wood essential oil (experimental; circle) conditions. CNT indicates the control condition, while EO indicates the experimental condition. Differences between conditions were not significant. Data are shown as mean ± SEM.

**Figure 8:**
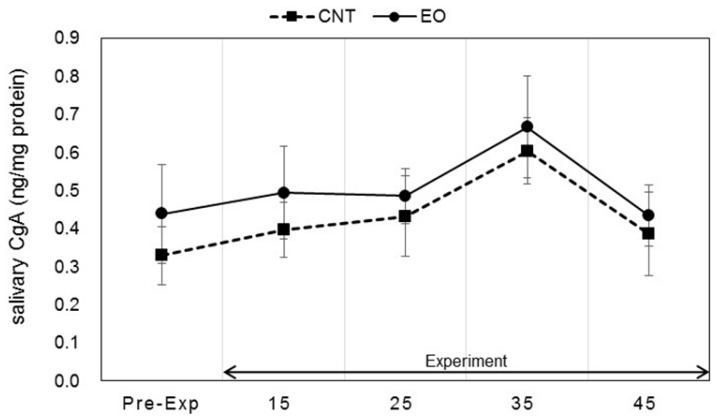
Variation of salivary CgA levels at pre- and during-experiment measurements. The plot in Figure 8 describes changes in the salivary CgA levels of participants in the control (square) and Japanese cedar wood essential oil (experimental; circle) conditions. CNT indicates the control condition, while EO indicates the experimental condition. Differences between conditions were not significant. Data are shown as mean ± SEM.

Salivary stress markers were measured before the participants entered the experiment room and after the work period as described in Figure 2. Statistically significant changes in salivary DHEA-s and IL-1β levels between conditions were observed. Each marker was measured before work and 15, 25, 35, and 45 min after work. The values of DHEA-s were 2.13 ± 0.47, 4.12 ± 0.56, 4.32 ± 0.44, 4.89 ± 0.43, and 4.79 ± 0.59 (ng/mL), respectively, in the Japanese cedar condition and 2.15 ± 0.33, 3.66 ± 0.33, 3.75 ± 0.55, 3.56 ± 0.40, and 3.53 ± 0.39 (ng/mL), respectively, in the control condition; the values at 35 min and 45 min were significant between the conditions (*p* < 0.01, *p* < 0.05 respectively). The differences between the values at pre-experiment and those after the working period were also significant (*p* < 0.01, *p* < 0.05 respectively) (Figure 4). The respective IL-1β values were 26.2 ± 11.8, 112.6 ± 39.1, 86.5 ± 17.9, 94.3 ± 21.6, and 102.1 ± 23.6 (pg/mL) in the Japanese cedar condition and 50.8 ± 21.0, 126.3 ± 50.8, 81.9 ± 26.2, 70.8 ± 24.3, and 78.6 ± 18.9 (pg/mL) in the control condition (Figure 5). The respective values of cortisol were 0.19 ± 0.03, 0.18 ± 0.04, 0.14 ± 0.02, 0.14 ± 0.03, and 0.15 ± 0.04 (µg/dL) in the Japanese cedar condition and 0.15 ± 0.03, 0.14 ± 0.02, 0.12 ± 0.01, 0.12 ± 0.02, and 0.12 ± 0.02 (pg/mL) in the control condition (Figure 6). The values of α-amylase were 51.4 ± 6.1, 43.5 ± 11.4, 58.4 ± 15.3, 58.6 ± 14.1, and 54.9 ± 14.5 (kIU/L, respectively, in the Japanese cedar condition and 65.1 ± 24.0, 57.4 ± 11.7, 81.9 ± 26.2, 89.8 ± 31.5, and 77.1 ± 15.3 (kIU/L), respectively, in the control condition (Figure 7). The values of CgA were 0.44 ± 0.13, 0.49 ± 0.12, 0.48 ± 0.07, 0.67 ± 0.13, and 0.43 ± 0.08 (ng/mg protein), respectively, in the Japanese cedar condition and 0.33 ± 0.08, 0.40 ± 0.07, 0.43 ± 0.11, 0.60 ± 0.09, and 0.39 ± 0.11 (ng/mg), respectively, in the control condition (Figure 8). No differences in the markers were observed between the conditions.

### 3.4. Arithmetic Work as a Monotonous Task

**Figure 9:**
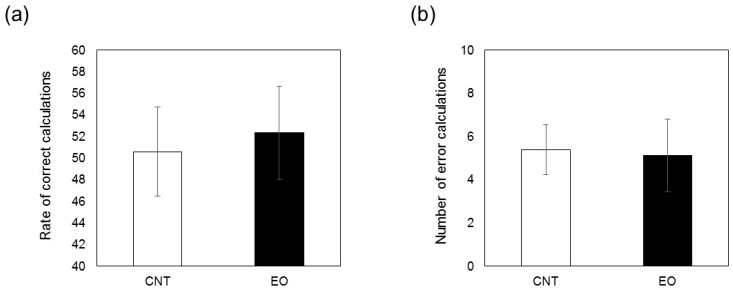
(**a**) Rate of correct calculations during arithmetic work. The bars represent the rate of correct calculations in the control (white) and Japanese cedar wood essential oil (experimental; black) conditions. CNT indicates the control condition, while EO indicates the experimental condition. Differences between both conditions were not significant. Data are shown as mean ± SEM; (**b**) Total number of error calculations during arithmetic work. The bars represent the total number of correct calculations in the control (white) and Japanese cedar wood essential oil (black) conditions. Differences between conditions were not significant. Data are shown as mean ± SEM.

Work performance was determined in terms of rate of correct calculations and total number of incorrect calculations; here, the rate of correct calculations was defined as the average total work time (Figure 9a,b). No difference in performance was found between the experimental and control conditions.

## 4. Discussion

Measurement of biomarkers in saliva is a noninvasive and convenient method to evaluate states of stress and fatigue; this method has been incorporated in various fields of interest, including psychiatry, sports medicine, and environmental and occupational health [26,27,28]. Changes in salivary biomarkers brought about by olfactory stimulation have been reported. Previous evaluations of salivary cortisol and CgA levels, for example, reveal that lavender and peppermint essential oils could be useful to relieve stress [29,30]. Yamaguchi et al. [31] also investigated the effects of fragrances on α-amylase activity, and Yoshizawa et al. [32] suggested that the essential oils of herbal medicine could potentially affect autonomic nervous activity by promoting changes in several biomarkers, including α-amylase, and cortisol. 

DHEA-s is a sulfated metabolite of DHEA, an androgen precursor secreted by the adrenal cortex. Several earlier studies demonstrate that acute psychosocial stress induces an increase in DHEA and DHEA-s concentrations, and hormones have been suggested to play a protective role against the potential damaging effects of excessive cortisol activity [33,34,35]. In our study, salivary DHEA-s levels increased and remained high throughout the rest period after arithmetic work only in the experimental condition; differences between groups observed in the rest period were significant (Figure 4). Li et al. [36] showed a significant increase in serum DHEA-s after asking participants to walk through a forest park, and the authors suggested that the stress status affects serum levels of DHEA-s. Hosoi et al. [37] indicated that long-term inhalation of fragrance could increase salivary DHEA levels, which could be related to the endocrine system. Several functions of DHEA-s, such as neuroprotective, anti-inflammatory, anti-glucocorticoid, and antidiabetic, have been suggested [38,39]. Oral administration of DHEA has been shown to activate immune functions [40]. In our study, levels of IL-1β as an index of immune functions showed a moderate increase only in the experimental condition about 35 and 45 min after the calculation task (Figure 5). Our findings indicate that increases in DHEA-s levels are induced by the volatile components of cedar wood essential oil, thereby suggesting the potential therapeutic benefits of the oil in DHEA secretion. Salivary cortisol levels at the pre-experimental period were the highest and did not increase during the experimental period in both groups (Figure 6). We thought it possible that some participants started our experiment in a psychologically anxious state based on the previous study [41].

Salivary α-amylase is an enzyme that is released from the salivary glands, while CgA is an acidic glycoprotein that is produced by the submandibular glands and secreted into saliva. The release of α-amylase and CgA is controlled by the sympathetic nervous system [42,43], and cardiac function is controlled by the autonomic nervous system. The results of this study showed no difference in the α-amylase and CgA levels of participants between the experimental and control conditions (Figure 7 and Figure 8). Previous studies have reported that the volatile compounds of Japanese cedar wood suppress activation of the sympathetic nervous system [18,20,22,44]. However, we could not clarify the effects of the volatile components of cedar wood essential oil on the indices of sympatho-adreno-medullary system in this study.

Work performance was determined by the number of per-minute correct calculations and total number of error calculations made in the total work period. No difference in work performance was found between the experimental and control conditions (Figure 9a,b). A previous study also reported no difference in work performance with or without Japanese cedar interior panels in a room [22]. The U-K test used in this study is widely used as a simple workload test that causes mental fatigue in participants. However, whether the participants’ feelings of stress varied depending on the amount and quality of work is not clear. We believe that the level of stress among participants was similar in both experimental situations.

Odor was subjectively evaluated using three questionnaires. Compared with that in the control condition, the VAS score of “natural” in the experimental condition was higher. However no significant differences in these questionnaires were found between the experimental and control conditions (Figure 3a–c). In this study, the concentration of Japanese cedar wood essential oil diffused into the experiment room was set to evoke comments of “comfortable” and “weak odor” based on a preliminary evaluation. This setting may explain the negligible difference in subjective evaluation of odor among the three questionnaires. 

We identified the VOCs emitted by Japanese cedar wood essential oil as δ-cadinene, 4-epi-cubebol, cubebol, and other sesquiterpenes, consistent with those identified in previous reports [45,46] (Table 1). The concentration of total volatiles was 161.5 µg/m^3^. Previous studies on indoor aromatherapy suggest that highly reactive substances could easily react with various pollutants in the air [47,48]; however, the concentration of terpenes in these studies was much higher than that determined in the present study. We suggest that the total volume and types of volatile organic compounds are important factors in producing comfortable and safe places for work and relaxation.

This study presents some limitations. First, the sample size used in the present study was fairly small and limited to only male participants. This limits the generalization of findings to include women and the general population. Second, the differences of time-course of DHEA-s secretion have not been clarified between the experimental and control conditions. In addition, the exact compounds of cedar wood essential oil that could affect increases in DHEA-s levels were not determined. We could not indicate an action mechanism of volatile compounds of cedar wood to the salivary DHEA-s levels in this study. Finally, we also did not collect data on the adverse effects of compounds of cedar wood essential oil, which is an important part of aromatherapy research.

## 5. Conclusions

In the present study, we found that Japanese cedar wood essential oil increased salivary dehydroepiandrosterone sulfate (DHEA-s) levels among males in the rest period after arithmetic work. Although other issues, such as differences brought about by gender and the contributions of each compound, must be thoroughly discussed, our findings indicate that the volatile organic compounds (VOCs) of Japanese cedar wood essential oil induce anti-stress effects during resting.

## Figures and Tables

**Table 1:** Chemical compounds obtained from Japanese cedar wood essential oil in this experiment.

RT (min)	Components	Composition (%)
26.9	*a*-Cubebene	0.8
28.2	Copaene	0.3
28.8	*b*-Cubebene	0.9
30.1	*b*-Caryophyllene	1.1
31.4	Muurora-3,5-diene	1.2
31.6	*a*-Humulene	1.0
32.4	Cadina-1(6),4-diene	2.8
32.5	*g*-Muurolene	0.3
32.7	Germacrene D	0.2
33.2	*cis*-Muurola-4(15),5-diene	4.5
33.4	Cubebol	16.4
33.5	*a*-Muurolene	6.5
34.0	*b*-Bisabolene	0.3
34.2	4-epi-Cubebol	18.0
34.4	*d*-Cadinene	21.4
34.5	(-)-Calamenene	1.3
34.5	*b*-Cadinene	2.1
34.9	Cubenene	1.4
35.6	*a*-Elemol	1.5
37.2	Gleenol	1.5
38.7	1,10-di-epi-Cubenol	5.8
38.9	*g*-Eudesmol	0.5
39.3	Epicubenol	3.8
39.5	*a*-Muurolol	1.1
39.7	*b*-Eudesmol	3.2
40.1	Dihydroeudesmol	0.3
42.5	Cryptomerione	0.7
51.1	Sandaracopimaridiene	0.2
55.2	Abietadiene	1.0
